# Strategic reminder setting for time-based intentions: Influence of metacognition, delay length, and cue visibility

**DOI:** 10.3758/s13421-025-01708-x

**Published:** 2025-04-16

**Authors:** Pei-Chun Tsai, Sam J. Gilbert

**Affiliations:** Institute of Cognitive Neuroscience, University College London, London, UK

**Keywords:** Cognitive offloading, Prospective memory, Metacognition, Metamemory

## Abstract

Time-based intentions, such as remembering to make a telephone call at a particular time or removing food from the oven after a delay, can be highly cognitively demanding. In everyday life, people often offload these demands to the external environment by setting alerts and reminders; however, this process of time-based intention offloading has rarely been examined experimentally. Here, we investigated this process in a paradigm where participants had to remember to press a key after a 10, 20, or 30 s delay, while simultaneously engaged in an ongoing two-back working memory task. Use of reminders improved accuracy, and participants were more likely to offload intentions at longer delays. This process was driven at least partially by metacognitive beliefs about the need for reminders, rather than the actual need. There was also an influence of time-monitoring demands: offloading was reduced when a clock was always visible, compared with a condition where participants had to press a button to reveal it. These results show that principles of cognitive offloading established in other domains also apply to time-based prospective memory: it improves performance, is influenced by cognitive demand, and guided by metacognitive beliefs.

Our ability to remember intentions after a delay, sometimes termed prospective memory (PM), plays a key role in everyday life (Cohen & Hicks, 2017; Kliegel et al., 2008; McDaniel & Einstein, 2007; Rummel & McDaniel, 2019; Scullin et al., 2015). Research into PM has often made a distinction between event- and time-based tasks (Einstein & McDaniel, 1990). An event-based PM task requires a person to perform an action when a particular event occurs—for example, pass on a message when one sees a particular colleague. By contrast, time-based PM tasks require an action at a particular time, for example take a cake out of the oven in 30 min. One difference between event- and time-based PM tasks is that time-based intentions are more likely to be triggered by self-initiated thoughts whereas event-based intentions may be triggered by external perceptual cues (Einstein & McDaniel, 1990; Kvavilashvili & Fisher, 2007).

While research into PM has traditionally focused on brain-based mechanisms for remembering intentions (e.g., McDaniel et al., 2015), recent studies have also investigated the ways in which people use their bodies and the wider physical environment (reviewed by Gilbert et al., 2023). This is a form of cognitive offloading (Risko & Gilbert, 2016) known as ‘intention offloading’ (Gilbert et al., 2023). For example, people might use the placement of physical objects, reminders such as to-do lists or diaries, or digital alerts to help them to remember intentions. Although real-world intention offloading may be particularly commonplace for time-based tasks (e.g., set a smartphone alert for 30 min), experimental studies of intention offloading have so far focused on event-based tasks instead (Boldt & Gilbert, 2019; Gilbert et al., 2020; Gilbert, 2015a, 2015b). Here, we investigate whether the principles established in previous research into intention offloading also apply to time-based tasks.

The first of the principles we examine here is that the decision to offload an intention onto an external reminder can be driven by a metacognitive belief that one might otherwise forget. Metacognition refers to the ability to monitor and regulate our own cognitive processes and abilities (Dunlosky & Metcalfe, 2008; Flavell, 1979; Fleming & Dolan, 2012; Koriat, 2007; Nelson & Narens, 1990). Previous research has emphasized the crucial role of metacognition in determining cognitive offloading strategies (Gilbert et al., 2020; Gilbert, 2015a, 2015b; Hu et al., 2019; Risko & Dunn, 2015), finding that people’s decision to set a reminder is influenced by their *beliefs* about whether they will remember, which may not necessarily correspond with their actual likelihood of remembering. Therefore, one aim of this study was to investigate whether metacognition also influences intention offloading strategies in a time-based task.

We also investigated the influence of two other factors that may influence time-based PM. The first of these was the duration over which the intention must be maintained. There is abundant evidence from research into retrospective memory that performance declines over a longer delay (Brown, 1958; Peterson & Peterson, 1959); however, the same does not necessarily apply to time-based PM. Einstein et al. (2003) compared PM accuracy between 5 s, 15 s, and 40 s delay periods, finding no significant decline across this range. However, their task was more appropriately classified as event- rather than time-based. Conte and Mcbride (2018) compared delays of 1, 3, and 6 min in both an event- and a time-based task. While event-based PM was reduced by longer delays, there was no decline in time-based PM. In sum, there is little evidence that longer delays harm time-based PM accuracy (though see Laera et al., 2023, for meta-analytic evidence that age-related impairment may be particularly pronounced at short intervals). Nevertheless, longer delays might increase the rate of intention offloading for two reasons. First, participants might believe that longer delays harm time-based PM, even if this is not true, leading to an increased offloading rate. Second, participants might prefer to offload intentions as a means of avoiding cognitive effort (Sachdeva & Gilbert, 2020), which would be exacerbated over a longer delay period.

A second factor we investigated (in Experiment 2) was the visibility of an external clock. The literature on time-based PM (Einstein et al., 1995; Harris & Wilkins, 1982; Henry et al., 2004; Mäntylä et al., 2009; Maylor, 1990) has consistently suggested that time-monitoring behaviour is important for successful performance. When a clock is constantly displayed, it acts as an external cue to prompt retrieval of the intention, thereby reducing the cognitive load of PM tasks. It also allows for easy checking of the time without additional cognitive or physical effort. On the other hand, when the clock is out of sight, clock-checking must be self-initiated, which is cognitively demanding and interrupts ongoing activities (Harris & Wilkins, 1982). Clock-checking behaviour is positively related to successful time-based PM (Mioni et al., 2020) and may occur strategically, with increasing frequency of clock checking as the target time approaches (Mioni & Stablum, 2014). Previous research has demonstrated that PM performance is worse with a hidden clock compared with a constantly displayed clock (Aberle et al., 2010; Mioni et al., 2020). Therefore, it might also be expected to trigger intention offloading behaviour.

To investigate the issues outlined above, we designed two experiments. In the first experiment, participants fulfilled time-based PM intentions after different lengths of short delays (10 s, 20 s, and 30 s). In half of the trials, participants performed the task unaided, while in the other half, they were permitted to set reminders. Before and after the experiment, participants provided a subjective rating of how well they expected they would perform or thought they had performed. The metacognitive judgements were given separately for different delay lengths. This allowed participants’ reminder setting to be related to objective performance measures and metacognitive judgements separately for different delay lengths.

The aim of the second experiment was to evaluate the relationship between reminder usage and time-monitoring behaviour, by manipulating clock visibility as a between-subject factor. One group of participants could always see a clock, the other had to press a button to reveal it.

We asked three main questions: 1) Do individuals offload time-based intentions more often when the delay is longer? 2) Do individuals offload intentions more often when time-monitoring is more cognitively demanding? 3) If these effects can be shown, can they be attributed, at least in part, to metacognitive factors? We predicted that the answer would be yes in each case. Me made two separate predictions for the influence of metacognition: first, that *between-subject* differences in individuals’ mean confidence would predict differences in reminder setting, and second that *within-subject* differences in confidence between conditions would predict differential rates of reminder usage. Before data collection, we preregistered our hypotheses, experimental procedure, and analysis plan for Experiment 1 (https://osf.io/snf8p/) and Experiment 2 (https://osf.io/jp462/).

## Experiment 1

### Methods

The design of this experiment was a 3 × 2 factorial in which the delay lengths (i.e., 10 s, 20 s, 30 s) and the offload conditions (unaided and reminder) were manipulated within subjects. In all conditions, participants performed an ongoing two-back working memory task. In this task, stimuli were letters of the alphabet. One letter at a time was presented at the middle of the screen. Participants responded to each stimulus by pressing the *X* key for targets (20% of trials) which were letters identical to the one presented two trials back, or by pressing the *Z* key for nontargets. The task was self-paced, with each letter remaining on-screen until a response was made, after which there was a 300-ms blank screen until the next letter was presented.

**Fig. 1:**
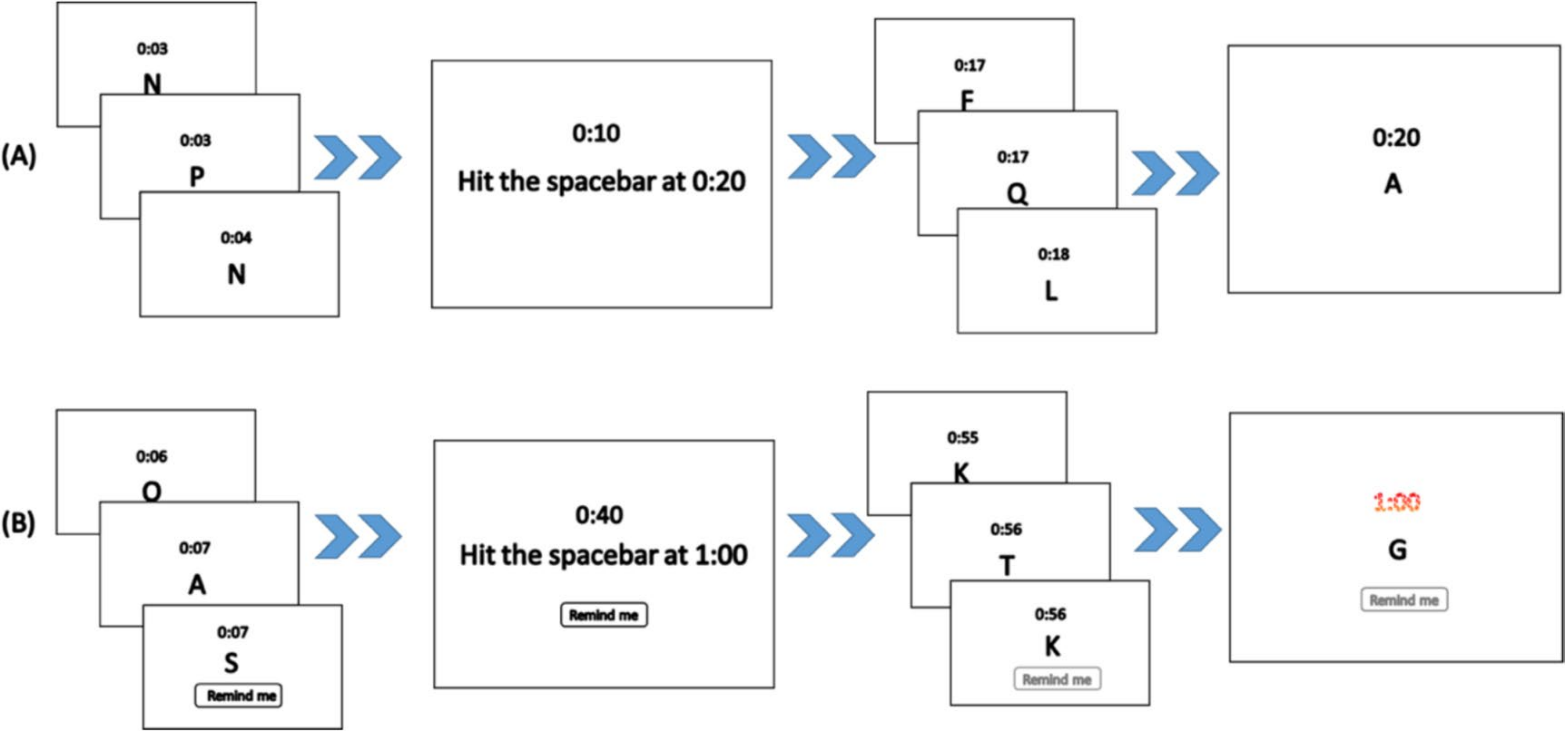
A schematic illustration of the PM task with a 20-s delay in the unaided and reminder conditions. *Note*. A digital clock above the letters starts running when the participants begin a block. The participants are instructed to perform the two-back task, and then the screen shows the instruction of a PM task. (**A**) Unaided condition: The participants should keep performing the two-back task. When the clock reaches the designated time they should remember to push the space bar. (**B**) Reminder condition: After the instruction of the PM task is shown, the participants could choose to click the button which shows ‘remind me’. They should keep going with the two-back task. The clock will flash to remind the participants when it is nearly the designated time

A digital clock was displayed above the letters, which started running when participants began the two-back task (see Fig. 1). During the task, the clock would periodically stop and participants would be presented with a message such as ‘Hit the space bar at 0:40’. The instructed time was always 10 s, 20 s, or 30 s after the current time. Participants could then press the space bar to resume the two-back task, which also restarted the clock. They were told that they had to press the space bar again within 2 s of the instructed time (e.g., 0:38 to 0:42). If they did so, the clock briefly flashed green to indicate that they had fulfilled the instruction. Following a 10-s delay from the previous instructed time, the clock was again stopped and a new instruction was presented. A single block consisted of a total of 12 time-based intentions (four repetitions of each delay length), presented in random order, with a total duration of 365 s.

To reduce the chance of ceiling performance on the PM task, the instructions before the experimental trials emphasised the importance of the two-back task (‘It is more important that you pay attention to the letters than the clock. But you should try to do the timer task at the same time, if you can’). We also encouraged participants to attend to the two-back task by paying an additional bonus to participants scoring in the top 50% of the two-back task, based on the total number of correct responses. Seeing as the task was self-paced this incentivised both speed and accuracy.

There were six blocks in the experiment, three blocks of the unaided condition and three blocks of the reminder condition. Therefore, there were a total of 72 PM instructions in the experiment. In the unaided condition, participants depended on their own memory to perform the PM task. In the reminder condition, they could set reminders by clicking a button labelled ‘remind me’. If they clicked this button, the clock would flash red during the 4-s window for PM responses, to indicate that it was now time to press the space bar. It was completely up to the participants whether to use the button or not. The small physical effort of moving the mouse pointer to the ‘remind me’ button and clicking it, along with the effort of moving a hand to the mouse and back again if both hands were resting on the keyboard, served as a disincentive against setting reminders if they were completely unnecessary. The blocks of the two conditions (unaided and reminder) alternated, and the starting condition was randomised across the participants.

#### Procedure

Participants first had a short practice session of the two-back task. Then the instructions for the PM task were explained, and participants were given a practice trial consisting of three PM instructions, each with a 10 s delay. This was then repeated with practice trials containing 20 s and then 30 s delays. Participants were required to repeat these trials if there was no correct PM response. Following each practice trial, participants were asked to make a prediction of the percentage of PM instructions they would successfully fulfil with a delay of that length. Therefore, separate predictions were made for 10 s, 20 s, and 30 s delays. Following this, the instructions for reminder setting were explained, and participants practiced setting a reminder. Then the main experimental trials began. Last of all, participants were asked to make retrospective confidence judgements (i.e., what percentage of instructions they had successfully fulfilled), separately for the three delays.

#### Participants

Participants were recruited via the Prolific website and took part by accessing a web-link that was provided to them. The study was approved by the UCL Research Ethics Committee (1584/002), and informed consent was obtained from all participants. They were paid £7 for taking part, plus an additional bonus of £1 if their performance of the two-back task was in the top half.

A priori power analysis was conducted based on a previous event-based PM study corresponding to the main investigation of the current study, which is evaluating the relationship between individual differences in metacognitive evaluation and reminder usage. Kirk et al. (2021) reported a correlation coefficient between a metacognitive measure and reminder usage of − 0.34. To achieve 80% power to replicate this effect (one-tailed, alpha = 0.05), a sample size of 52 was required (G*Power 3.1). We therefore aimed for a final sample of 52 participants, so that the study was powered for the key hypothesis-testing analyses of the correlation between confidence measures and reminder usage. Other analyses should be considered as exploratory, seeing as they were not subject to a formal power analysis. If participants were excluded due to the preregistered criteria below, they were replaced until the full sample of 52 was reached. The exclusion criteria were designed to ensure that participants attended sufficiently to the ongoing two-back task to achieve a minimum level of performance and to exclude participants with highly unusual performance of the PM task.

#### Exclusion criteria

- Accuracy below 70% on nonmatch trials of the two-back task.
- Accuracy below 40% on match trials of the two-back task.
- PM accuracy below 10%.
- PM accuracy more than 2.5 median absolute deviations from the median of all participants (Leys et al., 2013).
- PM confidence more than 2.5 median absolute deviations from the median of all participants (Leys et al., 2013).

There were 33 men and 19 women participants with a mean age of 26.4 years (*SD* = 8.8, range: 18–60). Participants took on average 70 min (minimum = 52 min, maximum = 4 h 11 min. NB: This includes any breaks between experimental blocks before resuming the task later; these breaks were unrestricted to ensure that participants could wait until they were free from distractions before resuming the task). Eleven participants were excluded due to our preregistered criteria (https://osf.io/snf8p/). Nine were removed due to accuracy below 70% on nonmatch trials of the two-back task. Two participants were excluded because their median PM confidence exceeded the 2.5 median absolute deviation criterion. No participant met any of the other exclusion criteria.

#### Dependent measures

A. These measures were calculated separately for each offload condition (unaided/reminder) as well as each delay length (10 s/20 s/30 s):
  i. PM accuracy (PM_any_): The mean proportion of PM trials where the space bar was pressed within the 4 s instructed response window.
  ii. PM accuracy restricted to the first response (PM_first_): Mean proportion of PM trials where the first space-bar press after the PM instruction occurred within the 4 s response window. This measure prevented participants from fulfilling the intention simply by pressing the space bar repeatedly throughout the delay period until a PM ‘hit’ occurred, without needing to remember the designated time. On a trial where the participant pressed the space bar at least once before the designated time, as well as during the instructed response window, this would count as a successful PM response according to the PM_any_ measure but not the PM_first_ measure.
  iii. False alarms: The mean number of times participants pressed the space bar outside the instructed time.
  iv. Misses: The proportion of trials where the space bar was not pressed at all.
B. These measures were calculated separately for each delay length (10 s/20 s/30 s):
  i. Reminder usage: The proportion of trials that participants set reminders when they were able to.
  ii. Confidence: The average of the pretask and posttask confidence ratings about PM accuracy in the unaided condition. The rationale for averaging pre- and post-task ratings was that the metacognitive beliefs measured by both ratings might be relevant to participants’ reminder setting strategies. The prospective rating might be particularly sensitive to initial beliefs that participants use while deciding on a strategy for the task, while the retrospective rating could also be sensitive to the way that participants update their beliefs during the experiment, which could lead to a shift in reminder-setting strategies. We decided to average these into a single confidence measure that could reflect all relevant information while reducing measurement error and the chance of false positives due to multiple comparisons.
  iii. Metacognitive bias: The difference between the subjective confidence and the actual accuracy (i.e., PM_first_).
    $$\text{Metacognitive bias}=\text{Confidence}-{\text{PM}}_{\text{first}}$$

Alongside these dependent measures, we extracted additional measures to investigate performance of the ongoing two-back task. These measures and relevant statistical analyses are reported in the Appendix.

#### Study materials

A demonstration of the full experiment including all instructions and practice can be viewed at https://cognitiveoffloading.net/PT1demo and analysis code is available at https://osf.io/snf8p/.

### Results

**Fig. 2:**
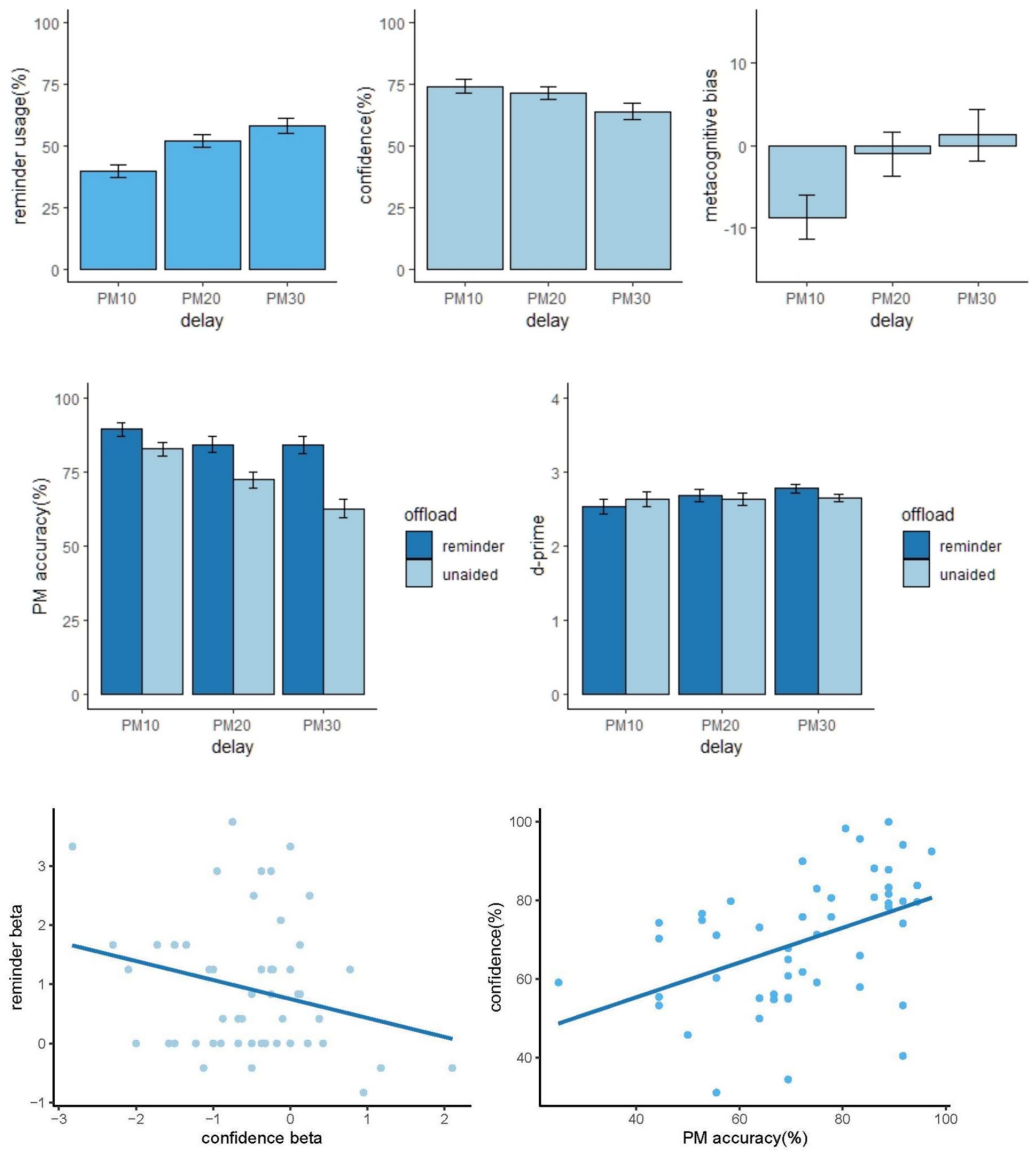
Results from the PM task and ongoing task in Experiment 1. *Note *Upper row: The error bars represent between-subject standard error. Middle row: Error bars represent within-subject confidence intervals for the comparison between reminder and unaided conditions, such that nonoverlapping bars indicate *p* < .05. Bottom left: Relationship between confidence beta (i.e., the relationship between confidence and delay) and reminder beta (i.e., the relationship between reminder usage and delay). Both confidence and reminder beta values were obtained by calculating a separate regression analysis for each participant. The blue line indicates linear regression for the relationship between the two betas across all individuals. Right: Relationship between confidence (averaged across the three delays) and PM_first_ (averaged across the three delays). The blue line indicates linear regression for the relationship between the two variables across all individuals. (Colour figure online)

All analyses were conducted in accordance with the preregistered plans, except where stated. All data analyses were conducted using R (Version 4.1.2; R Core Team, 2021). Regression analyses were performed by fitting linear models with R’s ‘lm’ function. See Table 1 and Fig. 2 for a summary of results. Participants performed an average of 2336 trials (range: 1638 – 2993) in the self-paced ongoing two-back task.

**Table 1:** Average scores of the PM measures for each delay length and analyses of variance examining the effects of delay length on each score (*F *values, significance levels and partial eta-square); standard errors of mean are shown in parentheses. ** = *p* < .01; *** = *p* < .001

Measure	Delay			*F*	*p*	η_*p*_^2^
	10 s	20 s	30 s			
PM_any_ (%)	87.2 (13.7)	86.7 (13.7)	85.4 (16.5)	.61	.544	.01
PM_first_ (%)	82.7 (15.6)	72.3 (17.8)	62.7 (23.1)	38.39	< .001^***^	.43
False alarms	1.42 (1.87)	3.65 (3.69)	7.04 (6.46)	34.97	< .001^***^	.41
Misses (%)	1.00 (1.41)	.85 (1.23)	.77 (1.22)	1.25	.291	.02
Confidence (%)	74.1 (19.8)	71.3 (16.1)	64.0 (18.1)	14.90	< .001^***^	.23
Metacognitive bias (%)	− 8.64 (19.5)	− .94 (19.4)	1.29 (22.5)	7.42	.003^**^	.12
Reminder usage (%)	39.7 (39.6)	52.1 (39.8)	58.0 (39.4)	24.10	.002^**^	.13

The calculation of the measures except reminder usage only includes the PM trials in the unaided condition

#### Effect of delay length

We first examined the effects of delay length on PM performance (PM_any_, PM_first_, false alarms and misses), confidence, metacognitive bias, and reminder usage (Table 1). Longer delays had no significant effect on PM_any_ accuracy or misses (F(2,102) < 1.3, p > 0.29, η^2^_*p*_ < 0.03), but led to increased false alarms and a decrease in PM_first_ accuracy (F(2,102) > 34, p < 0.001, η^2^_*p*_ > 0.40). Confidence scores declined across the delays (F(2,102) = 14.9, p < 0.001, η^2^_*p*_ = 0.23). There was also an effect on metacognitive bias: participants were underconfident at the shortest delay but this effect was diminished at the longer delays (F(2,102) = 7.42, p = 0.003, η^2^_*p*_ = 0.12). Reminder usage was significantly higher at longer delays (F(2,102) = 24.1, p = 0.002, η^2^_*p*_ = 0.13).

#### Metacognitive judgement and reminder usage

To examine whether individual differences in metacognitive judgement influenced individual differences in reminder setting, we calculated scores for confidence, reminder usage, unaided PM accuracy, and metacognitive bias (i.e., difference between confidence and accuracy), averaging across three delay lengths for each participant. We performed one-tailed Pearson correlation analyses based on our prediction of a negative relationship between reminder usage and each of these variables. However, none of these correlations was significant (confidence: *r*(50) =—0.08, *p* = 0.277; metacognitive bias: *r*(50) = 0.01, *p* = 0.536; PM_any_: *r*(50) =—0.05, *p* = 0.374; PM_first_: *r*(50) = 0.10, *p* = 0.250). Therefore, these findings do not support the hypothesis that less confident participants would use more reminders or that participants with poorer PM performance would use more reminders, at least when collapsed across the three delay lengths.

While collapsing over the three delays helps to reduce multiple comparisons, it may also obscure any effects that operate in a delay-specific manner. We therefore performed additional analysis to evaluate the impact of metacognitive judgement on reminder usage across different delays. As outlined in our preregistered analysis, we planned to perform separate regression analyses for each participant. We proposed in our preregistered plan to conduct a separate regression for each participant, looking at the relationship between confidence at the three durations (averaged across the two judgements) and reminder usage at the three durations. We predicted that a one-sample t test on the resulting beta values would show a significant negative relationship. Although this produced a significant result (*t*(48) = 2.7, *p* = 0.005, one-tailed as specified in the original pre-registration), we realised that this effect could be explained, trivially, if participants merely had lower confidence at longer delays and used more reminders at longer delays. This does not provide convincing evidence for a metacognitive influence on reminder setting. Therefore, we also took a more conservative non-preregistered approach. For each participant, we conducted *two* linear regressions, investigating A) the relationship between delay duration and confidence, and B) the relationship between delay duration and reminder setting. We then analysed the Pearson correlation between the resulting beta values, two from each participant. This provides a more conservative test of the preregistered hypothesis that declining confidence should predict increased reminder usage. There was a significant negative correlation (*r* (50) =—0.25, *p* = 0.037, one-tailed as in the analysis above because we preregistered a clear directional hypothesis that declining confidence should predict increasing reminder usage). This shows that participants who reported a steeper drop in confidence at longer delays, also tended to increase reminder usage more sharply at longer delays.

We conducted analogous analyses to evaluate the relationship between PM performance and confidence at the three delays. There was no significant effect for either PM_any_ (*r*(50) =—0.06, *p* = 0.346) or PM_first_ (*r*(50) = 0.20, *p* = 0.918). Therefore, the decline in confidence at longer delays did not predict the decline in accuracy, but it *did* predict the increase in reminder usage. This provides evidence for a metacognitive influence on reminder setting.

#### PM performance improvement

In the next analysis, we assessed the effect of offloading intentions by comparing PM performance between the unaided and reminder trials (see Fig. 2 Middle Row). Specifically, repeated-measures 2 × 3 ANOVAs on the measures of PM performance (i.e., PM_any_, PM_first_, false alarms, misses) with factors Offload (unaided, reminder) and Delay (10 s, 20 s, and 30 s) revealed that setting reminders significantly improved PM_any_ accuracy (Offload: *F*(1, 51) = 22.22, *p* < 0.001, η^2^_*p*_ = 0.3; Delay × Offload: *F*(2,102) = 2.57, *p* = 0.081, η^2^_*p*_ = 0.05) and PM_first_ accuracy (Offload: *F*(1,51) = 39.42, *p* < 001, η^2^_*p*_ = 0.44; Delay × Offload: *F*(2,102) = 14.95, *p* < 0.001, η^2^_*p*_ = 0.23), reduced false alarms (Offload: *F*(1,51) = 30.35,* p* < 0.001, η^2^_*p*_ = 0.37; Delay × Offload: *F*(2,102) = 19.44, *p* < 0.001, η^2^_*p*_ = 0.28), and misses (Offload: *F*(1,51) = 8.37,* p* = 0.006, η^2^_*p*_ = 0.14; Delay × Offload: *F*(2,102) = 0.08, *p* = 0.923, η^2^_*p*_ < 0.01). The effect was more prominent at longer delays for all measures except misses. This could potentially be explained by the finding that offloading was more common at longer delays. In general, the results showed that offloading intentions can improve PM performance.

#### Additional nonpreregistered analyses

First, two-tailed Pearson correlations showed confidence (averaged across delays) was correlated significantly with unaided PM performance (averaged across three delays) (PM_any_: *r*(50) = 0.37, *p* = 0.007; PM_first_: *r*(50) = 0.47, *p* < 0.001, see Fig. 2 Lower Row), as well as negatively correlated with false alarms (*r*(50) =—0.48, *p* < 0.001). Therefore, participants had some metacognitive insight into their PM performance, including both hits and false alarms.

Second, we tested whether a relationship between the amount of reminder usage and confidence may have been obscured by a distinct group of participants who never set reminders. After restricting the analysis to participants who set a reminder on more than one trial (42 out of 52 participants), a significant negative correlation was found between confidence and reminder usage (*r*(40) =—0.38, *p* = 0.006).

Finally, as requested by an anonymous reviewer, we performed a linear regression predicting reminder usage (averaged across delays) from PM accuracy and confidence. Neither of these factors was significant, regardless of whether we used the PM_any_ or PM_first_ measures, or whether we averaged prospective and retrospective confidence or considered them individually (t(49) < 0.72, p > 0.47).

### Discussion

Experiment 1 found that participants' PM performance decreased as the delay increased, but they compensated for the decline by offloading intentions more frequently. Importantly, to the extent that participants predicted worse performance at longer delays, they also set more reminders at longer delays. This was despite there being no relationship between the predicted decline in performance and actual decline. Therefore, results suggested that participants adjusted their intention offloading based on a metacognitive assessment of unaided memory ability, in line with previous research with event-based tasks (e.g., Boldt & Gilbert, 2019; Gilbert et al., 2020). Results also suggested that participants with lower average confidence were more likely to set reminders, but this effect was only found in a non-preregistered analysis restricted to participants who set more than one reminder.

PM performance was significantly improved when participants were given the option to offload intentions. Furthermore, the benefits of reminders was more pronounced at longer delays. This can be attributed to the increased usage of reminders at longer delays, facilitating participants’ ability to remember intended actions.

## Experiment 2

Experiment 2 aimed to further look into the effect of delay length and metacognition on intention offloading. We also evaluated how time-monitoring behaviour affects intention offloading, by comparing a condition where the clock was always visible (as in Experiment 1) with a condition requiring participants to press a button to reveal it.

### Methods

The design of this experiment was a 3 × 2 × 2 factorial in which the delay lengths (i.e., 10 s, 20 s, 30 s) and the offloading conditions (unaided and reminder) were varied within subjects, and the clock revealability was varied between subjects.

The overall experiment was identical to Experiment 1 except for a few changes. First, participants were explicitly told to press the space bar only once for each PM task and they were told that only the first space bar response for each PM task was counted, which discouraged participants from pressing the space bar repeatedly if they forgot the instructed time. Second, participants were randomly assigned to one of two groups, persistent-clock and hidden-clock. For the persistent-clock group, a digital clock was displayed above the letters in the same way as Experiment 1. For the hidden-clock group, participants could reveal the clock for 2 s by pressing the M key, otherwise no clock was displayed except for when participants were instructed about the next PM target time, so that they knew how long to wait. The last difference between Experiment 1 and Experiment 2 was that participants set reminders by clicking a button five times instead of once. The increased number of clicks helped to prevent a ceiling effect in reminder use. It also meant that setting a reminder incurred a greater physical cost than checking the hidden clock. Without this, setting a reminder with one mouse-click would incur comparable effort to checking the clock with a keypress. In this case there would be little reason for participants in the hidden-clock condition to ever use the clock-check mechanism rather than simply relying on a reminder. The button showed “remind me” and a number indicating the number of remaining clicks until the alarm was activated.

In all other ways, the procedure matched that of Experiment 1.

#### Participants

Participants were recruited via the Prolific website and took part by accessing a web-link that was provided to them. The study was approved by the UCL Research Ethics Committee (1584/002), and informed consent was obtained from all participants. The experiment took approximately one hour, and participants received a base payment, £7. To reduce the chance of ceiling performance on the PM task, we encouraged participants to attend to the 2-bask task by giving them an additional bonus. Participants were told they got one point for each correct response in the two-back task. If their scores were in the top half, they received an extra £1.

In Experiment 1, 42 out of 52 participants used reminders more than once. For these participants, there was a significant negative correlation coefficient between confidence and reminder usage (*r* = −0.35). To achieve 80% power to detect an effect of this size (one-tailed, alpha = 0.05), a sample size of 48 would be required. We therefore set a target sample size of 48 in each of the two groups (persistent-on and hidden-clock), i.e., 96 in total. Any participants who met the exclusion criteria were replaced until a final sample size of 96 was reached.

#### Exclusion criteria

Exclusion criteria were identical to Experiment 1, along with an additional criterion that we excluded participants where reminder usage across all PM tasks was equal to zero. This was based on the results from Experiment 1, suggesting that participants who never set reminders may form a distinct group, obscuring the relationship between reminder-usage and metacognitive beliefs amongst those that at least sometimes set reminders.

A total of 144 volunteers (71 persistent-on, 73 hidden-clock) were tested to reach the sample of 96 participants (48 participants in each group). Fourteen (2 persistent-on, 12 hidden-clock) were removed due to accuracy below 70% on non-match trials of the two-back task. Eight (3 persistent-on, 5 hidden-clock) were removed due to accuracy below 40% on match trials of the two-back task. Eighteen (13 persistent-on, 5 hidden-clock) were removed because they never used reminders. One participant (1 hidden-clock) was excluded because their mean PM accuracy was outside 2.5 median absolute deviations. Five participants (3 persistent-on, 2 hidden-clock) were excluded because their PM confidence was outside 2.5 median absolute deviations. No participant met any of the other exclusion criteria.

In the persistent-on group, there were 32 male and 16 female participants with a mean age of 27 years (*SD* = 8.2, range 18–56). In the hidden-clock group, there were 20 male and 28 female participants with a mean age of 26 years (*SD* = 6.2, range 18–48). Participants took on average 74 min (minimum = 51 min, maximum = 7 h 52 min) in the persistent-on group while participants took on average 59 min (minimum = 53 min, maximum = 1 h 55 min) in the hidden-clock group.

#### Dependent measures

We collected the same dependent measures as Experiment 1, except that we only considered PM_first_ as a measure of PM success. As set out in our pre-registration, this is because participants were now explicitly instructed that only their first response on each trial would be considered, therefore we picked a measure of PM accuracy to match this instruction.

In addition, we calculated a measure of clock checking for the hidden-clock group. This was done by dividing each PM delay period into successive 5-s intervals and counting the number of clock checks within each interval.

#### Study materials

A demonstration of the full experiment including all instructions and practice can be viewed at https://cognitiveoffloading.net/PT2demo and analysis code is available at https://osf.io/jp462/

### Results

**Fig. 3:**
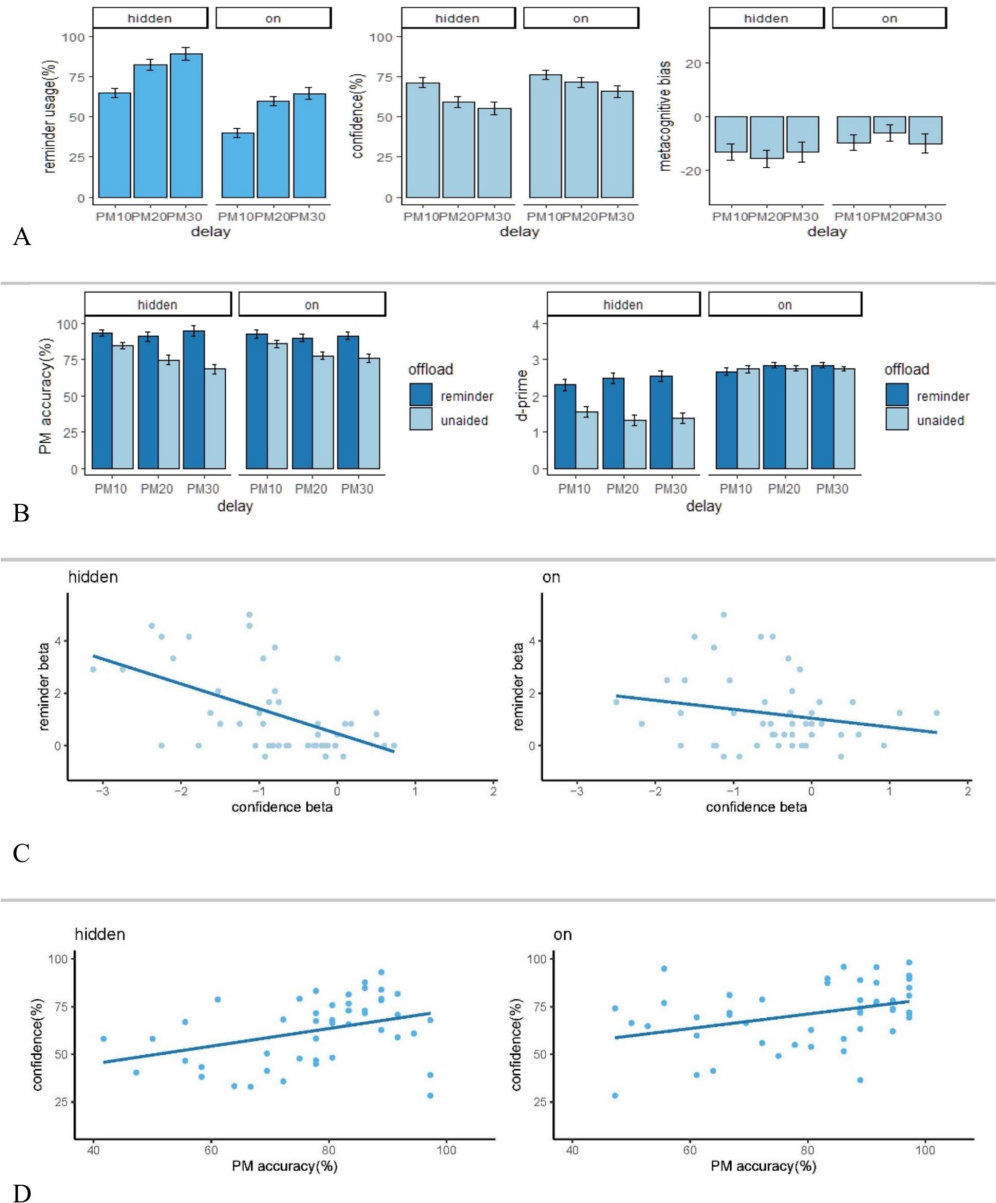
Results from the PM task and ongoing task in Experiment 2. *Note ***A** Error bars represent between-subject standard error. **B** Error bars represent within-subject confidence intervals for the comparison between reminder and unaided conditions such that nonoverlapping bars indicate *p* < .05. **C** Relationship between confidence beta (i.e., the relationship between the confidence and delay) and reminder beta (i.e., the relationship between the reminder usage and delay). Both confidence and reminder beta values were obtained by calculating a separate regression analysis for each participant. The blue line indicates linear regression for the relationship between the two variables across all individuals. Left: the hidden-clock group. Right: the persistent-on group. **D** Relationship between confidence (averaged across the three delays) and PM performance (averaged across the three delays). The blue line indicates linear regression for the relationship between the two betas across all individuals. Left: the hidden-clock group. Right: the persistent-on group. (Colour figure online)

All analyses were conducted in accordance with the preregistered plans, except where stated. All data analyses were conducted using R version 4.1.2 (R Core Team, 2021). Regression analyses were performed by fitting linear models with R’s ‘lm’ function. See Table 2, and Fig. 3 for a summary of results. Participants in the persistent-clock group performed an average of 2,245 trials (range: 1,085–2,994) in the self-paced ongoing two-back task. The equivalent figures for the hidden-clock group were 2,363 (range: 1,,043–3480).

**Table 2:** Average scores of the PM measures for each delay length and analyses of variance examining the effects of clock, delay, and the interaction on the measure scores (*F *values, significance levels and partial eta-square); standard errors of mean are shown in parentheses. ** = *p* < .01; *** = *p* < .001

	Hidden-clock group			Persistent-on group			Clock		Delay		Clock × Delay	
	Delay			Delay								
Measure	10s	20s	30s	10s	20s	30s	F	η_*p*_^2^	F	η_*p*_^2^	F	η_*p*_^2^
PM_ANY_ (%)	84.4 (14.5)	74.7 (20.6)	68.4 (24.1)	85.8 (15.8)	77.4 (18.3)	75.9 (20.7)	1.44	.02	23.03***	.20	1.33	.01
False alarms	1.77 (2.22)	3.81 (4.89)	5.10 (5.74)	3.54 (17.7)	2.50 (3.31)	2.88 (2.86)	.31	< .01	.76	< .01	1.84	.02
Misses (%)	.50 ( .97)	.75 ( 1.10)	.75 ( 1.16)	.77 (1.02)	1.00 (1.32)	.88 (1.47)	1.44	.02	1.57	.02	.16	< .01
Confidence (%)	71.1 (17.0)	59.0 (19.2)	55.2 (22.2)	76.1 (17.1)	71.3 (16.0)	65.8 (21.1)	7.34 **	.07	39.14 ***	.29	3.23	.03
Metacognitive bias (%)	− 13.3 (20.7)	− 15.7 (23.0)	− 13.2 (25.9)	− 9.63 (20.8)	− 6.13 (20.5)	− 10.1 (25.0)	2.17	.02	.05	< .01	1.08	.01
Reminder usage (%)	64.8 (40.1)	82.1 (28.8)	89.1 (20.4)	39.9 (42.0)	59.7 (37.2)	64.4 (36.2)	13.47 ***	.13	53.22 ***	.36	.15	< .01

The calculation of the measures except reminder usage only includes the PM trials in the unaided condition

#### PM performance

We examined PM performance using a 3 × 2 mixed ANOVAs with clock (persistent-clock, hidden-clock) as the between-subjects variable and delay (10 s, 20 s, 30 s) as the within-subjects variable. The analysis revealed a significant main effect of delay, *F*(2, 188) = 23.03, *p* < 0.001, η_p_^2^ = 0.20, on PM accuracy, but no significant effect of clock nor Delay × Clock interaction: clock, *F*(1, 94) = 1.44, *p* = 0.233, η_p_^2^ = 0.02; Clock × Delay: *F*(2, 188) = 1.33, *p* = 0.268, η_p_^2^ = 0.01, indicating that PM performance declined over time but was not significantly influenced by clock revealability.

#### Reminder usage

However, a similar analysis on reminder usage showed both delay length and clock revealability had a significant effect (Delay: *F*(1.42, 133.06) = 53.22, *p* < 0.001, η_p_^2^ = 0.36; Clock: *F*(1, 94) = 13.47, *p* < 0.001, η_p_^2^ = 0.13; Delay × Clock interaction: *F*(1.42, 133.06) = 0.15, *p* = 0.785, η_p_^2^ < 0.01). Therefore, as predicted, participants set more reminders when the delay was longer or when the clock was not always visible. We also conducted a 3 × 2 ANOVA to examine metacognitive judgements and found that participants were significantly less confident (averaged across the pretask and posttask judgements) at longer delays, *F*(1.68, 158.2) = 39.14, *p* < 0.001, η_p_^2^ = 0.29, and when the clock was hidden, *F*(1, 94) = 7.34, *p* = 0.008, η_p_^2^ = 0.07. There was a trend towards increased influence of clock visibility at longer delays (or, equivalently, an increased influence of delay when the clock was not visible). However, the Delay × Clock interaction just missed significance, *F*(1.68, 158.2) = 3.23, *p* = 0.051, η_p_^2^ = 0.03.

Along with the analyses of confidence, we conducted analogous ANOVAs investigating metacognitive bias (i.e., difference between confidence and actual accuracy). There was no significant effect of delay or clock revealability on metacognitive bias (Delay: *F*(2, 188) = 0.03, *p* = 0.974, η_p_^2^ < 0.01; Clock: *F*(1, 94) = 2.17, *p* = 0.144, η_p_^2^ = 0.02; or Delay × Clock interaction: *F*(2, 188) = 1.08, *p* = 0.343, η_p_^2^ = 0.01).

#### Metacognitive judgement and reminder usage

We conducted a linear regression to evaluate the relationship between reminder usage (averaged across all delays) and metacognitive judgement with regressors: A) mean confidence (averaged across all judgements, and mean-centred), B) mean PM accuracy (averaged across all delays), and C) clock revealability (coded as persistent-on = 1, hidden-clock = − 1). The analyses of the beta coefficients for confidence and PM accuracy were one-tailed, based on the prediction that there should be a negative relationship between reminder use and each of the measures. Only clock revealability was a significant predictor of reminder usage (Confidence: *β* = 0.01, *p* = 0.953; PM accuracy: *β* = − 0.26, *p* = 0.257; Clock revealability: *β* = − 11.54, *p* = 0.001). A subsequent regression analysis was performed with regressors: A) mean confidence (averaged across all judgements, and mean-centred), B) clock revealability (coded as persistent-on = 1, hidden-clock = − 1), and C) Confidence × Clock Revealability interaction. The one-tailed analyses of beta coefficients showed that clock revealability was a significant regressor, along with the Confidence × Clock Revealability interaction (Confidence: *β* = − 0.11, *p* = 0.565; Clock revealability: *β* = − 11.46, *p* < 0.001; Confidence × Clock Revealability: *β* = − 0.42, *p* = 0.034). However, preregistered one-tailed Pearson correlations did not reveal a significant association between individuals’ average metacognitive judgement and reminder usage in either group: persistent-on group, *r*(46) = − 0.23, *p* = 0.053; hidden-clock group, *r*(46) = 0.20, no *p* value calculated seeing as the *r* value was positive.

We then repeated the above analyses using metacognitive bias (i.e., the difference between confidence and PM accuracy) instead of confidence. Similarly to the previous findings, we found that only clock revealability significantly predicted reminder usage (the first model: metacognitive bias: *β* = 0.013, *p* = 0.953; PM accuracy: *β* = − 0.25, *p* = 0.315; clock revealability: *β* = − 11.54, *p* = 0.001; the second model: metacognitive bias: *β* = 0.12, *p* = 0.515; clock revealability: *β* = − 15.56, *p* < 0.001; Metacognitive Bias × Clock Revealability: *β* = − 0.29, *p* = 0.116; one-tailed Pearson correlation: persistent-on group *p* = 0.285; hidden-clock *p* = 0.970).

Overall, the study did not provide significant evidence to support the hypothesis that individuals who generally exhibit lower levels of confidence or higher levels of underconfidence about unaided memory ability are more likely to set reminders.

#### Metacognitive judgement and effect of delay

We conducted simple linear regression analyses for each subject to evaluate the relationship between delay duration and confidence levels (averaged across the two judgements), as well as the relationship between delay duration and reminder usage. We observed a significant one-tailed Pearson correlation between these two beta values in the hidden-clock group, *r*(46) = − 0.53, *p* < 0.001, but this correlation did not reach significance in the persistent-on group, *r*(46) = − 0.21, *p* = 0.079. We conducted similar analyses to evaluate the relationship between PM performance and confidence across three delays: persistent-on group, *r*(46) = − 0.14, *p* = 0.172; hidden-clock group: *r*(46) = − 0.05, *p* = 0.375, as well as the relationship between PM performance and reminder usage across three delays: persistent-on group, *r*(46) = 0.18, *p* = 0.887; hidden-clock group: *r*(46) = − 0.15, *p* = 0.153. Like Experiment 1, these results provide evidence for a metacognitive influence on intention offloading: a drop in confidence across the 3 days predicted an increase in reminder setting, even though this decrease in confidence did not predict the decrease in accuracy.

#### PM performance

In the next analysis, we assessed the effect of offloading by comparing PM performance between unaided and reminder trials (see Fig. 3). Specifically, repeated-measures 2 × 3 × 2 ANOVAs on the measures of PM performance (i.e., PM_first_, false alarms, misses) were performed with within-subject factors offload (unaided, reminder) and delay (10 s, 20 s, and 30 s) as well as with a between-subject factor clock (persistent-clock, hidden-clock). The analysis revealed that the main effects of offload and delay were significant (Offload: *F*(1, 94) = 99.15, *p* < 0.001, η_p_^2^ = 0.51; Delay: *F*(1.78, 167.48) = 20.40, *p* < 0.001, η_p_^2^ = 0.18). However, the main effect of clock was not significant, *F*(1, 94) = 0.27, *p* = 0.607, η_p_^2^ < 0.01. There was also a significant interaction between Offload and Delay (*F*(2, 188) = 15.09, *p* < 0.001, η_p_^2^ = 0.14). As can be seen in Fig. 3B, the effect of offloading on the PM accuracy was more prominent at longer delay in both groups. The other interactions were not significant: Clock × Offload: *F*(1, 94) = 3.74, *p* = 0.056, η_p_^2^ = 0.04; Delay × Clock: *F*(1.78, 167.48) = 0.31, *p* = 0.707, η_p_^2^ < 0.01; Delay × Clock × Offload: *F*(2, 188) = 1.88, *p* = 0.155, η_p_^2^ = 0.02. Further analyses showed the Clock × Offload interaction was significant at 30, *F*(1, 94) = 6.05, *p* = 0.016, but not 10 s, *F*(1, 94) = 0.34, *p* = 0.564, or 20 s, *F*(1, 94) = 0.912, *p* = 0.342. Similar ANOVA analyses conducted on false alarms and misses showed the main effect of offload was significant: false alarms, *F*(1, 94) = 21.63, *p* < 0.001, η_p_^2^ = 0.19; misses, *F*(1, 94) = 25.32, *p* < 0.001, η_p_^2^ = 0.21. Thus, the results overall demonstrated that PM performance was improved by intention offloading, especially at longer delays, which is congruent with the findings of Experiment 1. Moreover, the hidden-clock group showed more prominent reminder effect on PM performance than the persistent-on group, but only at longer delays.

#### Additional, nonpreregistered analyses

**Fig. 4:**
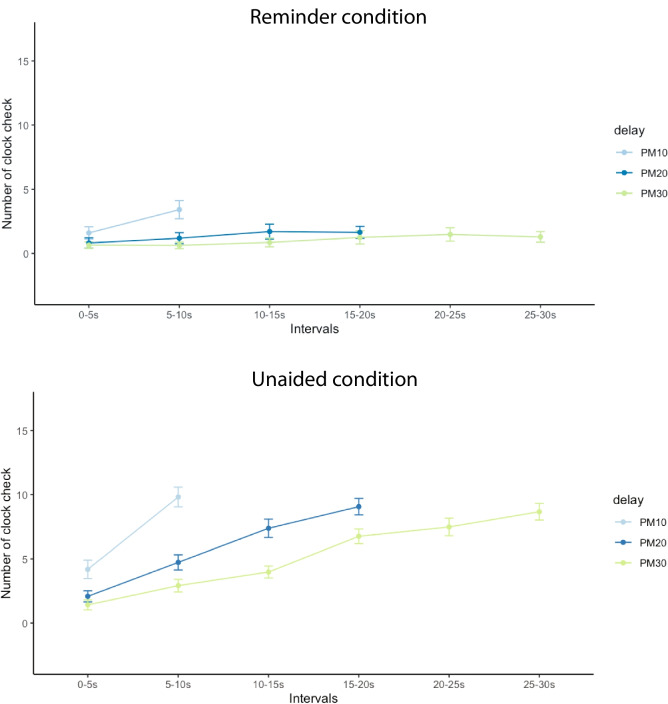
Total number of clock checks for the hidden-clock group at each 5-s interval following the PM instruction. Results are summed over the 12 trials corresponding to each condition, then averaged across participants. *Note.* Error bars represent between-subject standard errors. (Colour figure online)

This section reports some exploratory tests conducted in addition to the preregistered ones described above. First, we conducted a two-tailed one-sample *t* test on the metacognitive bias (averaged across the delay lengths), showing participants in both groups were significantly underconfident of the unaided memory performance: persistent-on, *t*(47) = 3.31, *p* = 0.002; hidden-clock, *t*(47) = 5.33, *p* < 0.001. Second, confidence (averaged across delays) was found to be significantly correlated with unaided PM accuracy (averaged across delays) in both persistent-on: *r*(46) = 0.37, *p* = 0.009, and hidden-clock groups, *r*(46) = 0.40, *p* = 0.005, indicating some metacognitive insight into unaided task performance. Finally, time monitoring frequency (averaged across delays) was positively correlated with unaided PM accuracy (averaged across delays) in the hidden-clock group: *r*(46) = 0.48, *p* < 0.001, consistent with prior research (Mioni & Stablum, 2014; Mioni et al., 2020). The pattern of the highest frequency for the latest interval was observed, but not when people were allowed to set reminders, likely due to waiting for the clock to flash (Fig. 4).

### Discussion

There were two main results from this experiment. First, as in Experiment 1, there was evidence for a metacognitive influence on intention offloading, because the drop in confidence across the three durations predicted the increase in reminder-setting, even though it was not related to unaided accuracy. There was also evidence for a second factor influencing offloading: participants were more likely to set reminders in the hidden-clock than the visible-clock condition. Therefore, the availability of an alternative external cue was related to intention offloading as well.

Performance of the PM task was relatively high (> 75%) in all conditions, even when the clock was hidden and offloading was not permitted. Therefore, over the brief retention intervals of up to 30 s, participants were generally capable of maintaining time-based intentions with little external support, despite their preference for reminder setting when this strategy was available.

Analysis of time-monitoring behaviour in the hidden-clock group showed that a higher rate of clock checking was associated with a greater likelihood of fulfilling time-based intentions. Results also showed that clock checking was most common in the interval immediately preceding the intended response, when participants remembered intentions without the help of external reminders. Both findings are consistent with previous evidence (Mioni et al., 2020). However, whereas previous studies have shown a sharp increase in clock-checking frequency just before the intended response (Mioni & Stablum, 2014; Mioni et al., 2020; Munaretto et al., 2022), the pattern in the present study appeared approximately linear. One potential explanation for this is that the retention interval in the present study (between 10 and 30 s) was shorter than that in previous studies (Mioni & Stablum, 2014; Mioni et al., 2020; Munaretto et al., 2022), which were on the order of minutes.

## General discussion

The current experiments aimed to investigate factors influencing intention offloading in time-based PM tasks, an issue that has received little prior experimental attention. We found that participants’ PM performance declined at longer delays, and they compensated for this by offloading intentions more frequently. However, increased reminder-usage at longer delays did not just reflect the need for such reminders, but also participants’ metacognitive judgements. We also found that intention offloading was increased in the absence of a visible clock. Therefore, participants tended to use reminders when A) they believed that the delay duration would impair their performance, and/or B) they needed to rely on internal time estimation rather than an external clock.

The current results, along with some event-based studies (Boldt & Gilbert, 2019; Gilbert, 2015b, Experiment 1b) demonstrated that people’s metacognitive judgements predicted their propensity to offload intentions while their objective unaided accuracy did not. Participants who sharply reduced their confidence at longer delays had a greater increase in reminder usage, but this drop in confidence did not reflect objective performance. Thus, on an individual level, people had a greater propensity to set reminders in the delay condition where they believed they could benefit more from the offloading strategy, irrespective of actual performance.

Given that participants did not demonstrate a tendency to adjust intention offloading based on actual performance across different delays, it is worth noting that individuals who performed more poorly and potentially benefited more from setting reminders did not increase the average number of reminders they set, despite having some awareness of unaided memory performance which was reflected in a significant correlation between an individual’s confidence (averaged across delays) and PM performance (averaged across delays). Moreover, individuals who predicted lower memory performance (averaged across delays) did not correspondingly increase the average number of reminders. Thus, although a metacognitive influence on intention offloading could be seen *within* individuals across the three delay conditions, there was little evidence of an impact of metacognitive variation *between* individuals on their offloading strategies. Given that between-participant variation in metacognitive judgements did significantly correlate with memory performance, this suggests a disconnect between individuals’ metacognitive knowledge (i.e., beliefs about cognitive ability) and metacognitive control (i.e., the use of those beliefs to influence strategy choices; see Boldt & Gilbert, 2022; Redshaw et al., 2018; Scarampi & Gilbert, 2020, for discussion).

### Time monitoring behaviour

Studies have suggested that the cognitive load of a PM task can be manipulated by controlling the visibility of a clock. The hidden clock group, which was considered to be under higher cognitive load, did not exhibit worse unaided PM performance compared with the persistent-on group. However, they did demonstrate worse performance of the ongoing task compared with the persistent-on group (see Fig. 3 and Appendix for further information). This can be seen as a ‘cost’ of the additional PM demands when the clock is not visible, akin to the PM costs observed by other authors measured as impaired ongoing task performance when PM demands are introduced (Joly-Burra et al., 2018; Smith et al., 2012).

Regarding the main question whether clock visibility influences reminder usage, our results were in line with prior event-based PM studies (Gilbert, 2015a; Scarampi & Gilbert, 2021), which have suggested that the higher cognitive load could lead people to set more reminders. As expected, participants in the hidden-clock group used more reminders than those in the persistent-on group. The linear regression analyses showed clock visibility contributed unique variance to reminder usage, independent of any variance contributed by confidence, suggesting that individuals in the hidden-clock group might set more reminders even if they had the same confidence as individuals in the persistent-on group. One possible explanation is that people set reminders not only to improve performance but also to reduce cognitive effort (Sachdeva & Gilbert, 2020), however this remains speculative seeing as we did not collect any explicit measure of perceived effort.

Setting reminders reduced cognitive load by reducing the need for internal time monitoring, however it also requires additional cognitive and physical effort due to the need to switch attention to the reminder button and press it (once in Experiment 1, five times in Experiment 2). Intention offloading can therefore be seen as requiring cost–benefit calculation (Chiu & Gilbert, 2023; Gilbert et al., 2020) or value-based decision making (Gilbert, 2024). It is likely that people rely on internal memory for easier tasks and use more reminders for tasks that require greater cognitive effort.

### Reminder benefit

PM performance was significantly improved when participants were allowed to offload intentions. The benefit of reminders was greater at longer delays. This may be explained in part by the fact that participants used more reminders at longer delays. Along with PM performance, another possible manifestation of the reminder benefit was the performance of the ongoing task. The current study showed that ongoing accuracy was improved by using reminders in the hidden-clock group, and the benefit was greater at longer delays (see Fig. 3 and the Appendix for further information). In contrast, the benefit for ongoing accuracy was not significant in the persistent-on group. Neither group showed a response-time benefit when using reminders. Therefore, results suggest that the benefits of reminder usage for an ongoing task may be particularly apparent with accuracy rather than speed measures.

### Limitations and future directions

The main methodological limitation of the present study was the brief delay duration used in the paradigm. Although the present study used delays in the short-term memory range of 30 s or less, most experimental studies have investigated longer intervals (Laera et al., 2023), and people often need to maintain intentions for longer durations in everyday time-based PM tasks. It remains uncertain whether people would offload intentions similarly in such tasks, though see Gilbert (2015a) for evidence that intention offloading tasks with brief delays in the range of seconds can predict everyday PM over a period of days. It is also unclear whether offloading strategies would be similar when participants are instructed to prioritise the PM task, rather than prioritising the ongoing task as participants were instructed in the present study. A promising approach for future research is to evaluate whether people in naturalistic settings adjust intention offloading based on metacognitive judgement, as they do in experimental tasks (see Scott & Gilbert, 2024, for initial evidence in support of this). It would also be useful to test whether the relationships between objective ability, metacognitive judgement, and strategic reminder setting, which in this study were all measured within a single experimental task, would also generalise across different tasks or cognitive domains (see Sachdeva & Gilbert, 2024, for discussion of this point). We note that a relatively high number of participants were excluded due to our preregistered exclusion criteria, so it is not known how far the results generalise to all individuals. On the other hand, the age range in these studies was relatively large, making these findings more representative of the general population than the convenience samples of undergraduate students that are often used in experimental psychology research (Wild et al., 2022).

Another promising approach for future research is to investigate the extent to which metacognitive interventions such as metacognitive training could improve individuals’ offloading strategy. Given that reminders are highly effective in both real-world (e.g., Jones et al., 2021) and laboratory (e.g., Gilbert et al., 2020) settings, and that metacognitive error may affect intention offloading strategies (Boldt & Gilbert, 2019; Gilbert et al., 2020; Gilbert, 2015b), there is potential for metacognitive interventions to optimise such strategies and hence support the fulfilment of real-world intentions.

## Conclusion

As stated in the Introduction, this study was motivated by three main research questions: 1) Do individuals offload time-based intentions more often when the delay is longer? 2) Do individuals offload intentions more often when time-monitoring is more cognitively demanding? 3) If these effects can be shown, can they be attributed, at least in part, to metacognitive factors? We found preliminary evidence that the answer is ‘yes’ for all three questions, although the influence of metacognition was only clearly seen on a within-subject and not a between-subject basis. Future research could test the generalisability of these findings to longer delays and naturalistic settings, as well as test the impact of metacognitive interventions on offloading strategies.

## Data Availability

Data and code to reproduce all analyses, along with the original study pre-registrations can be found at (Experiment 1: https://osf.io/snf8p/; Experiment 2: https://osf.io/jp462/). Study materials can be viewed at https://cognitiveoffloading.net/PT1demo and https://cognitiveoffloading.net/PT2demo

## Appendix

### Analysis of ongoing task performance

The measures on the ongoing task were calculated separately for each offload condition (unaided/reminder), as well as each PM target condition (no-target/10 s/20 s/30 s). The no-target condition refers to the periods when there was no current active PM intention. Before analysing data from the ongoing task, we excluded the following trials: a) the first trial of each block, b) the trial before and after any spacebar press, c) the trial after any PM instruction, and d) any trial with RT below 150 ms or above 3000 ms. The RT exclusions were intended to remove trials that were either so fast that the participant likely did not attend to the 2-back task, or so slow that they were likely distracted. The precise figures of 150 ms and 3000 ms were arbitrary but fixed before the data were collected. In experiment 2, we additionally excluded the trial before and after a clock-check response in the hidden-clock group. The following measures were extracted:

i. Accuracy of the match trials on the 2-back task: The mean target accuracy (i.e. proportion of the correct responses to the target letters) on the 2-back task
ii. Accuracy of the non-match trials on the 2-back task: The mean non-target accuracy (i.e. proportion of the correct responses to the non-target letters) on the 2-back task
iii. D-prime on the 2-back task(*d’*): The difference between the z-transforms of the two previous measures.
iv. Reaction time of the match trials on the 2-back task: The mean reaction time of the match trials on the 2-back task
v. Reaction time of the non-match trials on the 2-back task: The mean reaction time of the non-match trials on the 2-back task

### Analysis of ongoing task performance in Experiment 1

We investigated whether the performance of the ongoing task differed among three delay lengths and was affected by reminder usage (see Fig. 2). A repeated-measures ANOVA analysing the *d’* measure from the 2-back task with factors Offload (unaided, reminder) and Delay (no-target, 10 s, 20 s, 30 s) showed that the d-prime of the 2-back task was not significantly affected by offloading (*F*(1,51) = 0.15, *p* = 0.696, η^2^_*p*_ < 0.01) but there was a main effect of Delay (*F*(3,153) = 2.87, *p* = 0.04, η^2^_*p*_ = 0.05) with poorer performance at shorter delays (no-target: *M* = 2.65, *SD* = 0.76; 10 s: *M* = 2.53, *SD* = 0.77; 20 s: *M* = 2.63, *SD* = 0.79; 30 s: *M* = 2.68, *SD* = 0.77). The Offload x Delay interaction was not significant (*F*(2.53,129.01) = 2.20, *p* = 0.102, η^2^_*p*_ = 0.04). We also analysed the reaction time measure with factors Offload (unaided, reminder), Match (match, nonmatch), and Delay (no-target, 10 s, 20 s, 30 s). There were significant main effects of Delay (*F* (3, 153) = 15.23, *p* < 0.001, η^2^_*p*_ = 0.23), Match (*F* (1, 51) = 35.03, *p* < 0.001, η^2^_*p*_ = 0.41), and a Delay × Match interaction (*F* (2.03, 103.46) = 3.25, *p* = 0.042, η^2^_*p*_ = 0.06). The main effect of Offload (*F* (1, 51) = 0.55, *p* = 0.462, η^2^_*p*_ = 0.01) and other interactions (Delay × Offload × Match: *F* (2.45, 125.14) = 1.86, *p* = 0.150, η^2^_*p*_ = 0.04; Offload × Match: *F* (1, 51) = 0.65, *p* = 0.424, η^2^_*p*_ = 0.01; Offload × Delay: *F*(2.36, 120.48) = 0.76, *p* = 0.49, η^2^_*p*_ = 0.02) were not significant. Overall, results showed that performance of the 2-back task was not significantly affected by offloading.

### Analysis of ongoing task performance in Experiment 2

We investigated whether the performance of the ongoing task differed across the three delay lengths, and was affected by reminder usage in the two groups (see Fig. 3). Results of a repeated-measures ANOVA on the d’ measure showed that the main effects and interactions of within-subject factors, Offload (unaided, reminder) and Delay (no-target, 10 s, 20 s, 30 s), as well as a between-subject factor, Clock (persistent-on, hidden-clock) were all significant (Clock: *F* (1, 94) = 21.56, *p* < 0.001, η^2^_p_ = 0.19; Delay: *F* (2.5,234.9) = 26.60, *p* < 0.001, η^2^_p_ = 0.22; Offload: *F* (1, 94) = 61.19, *p* < 0.001, η^2^_p_ = 0.39; Clock × Delay: *F* (2.5, 234.9) = 15,72, *p* < 0.001, η^2^_p_ = 0.14; Clock × Offload: *F* (1, 94) = 57,14, *p* < 0.001, η^2^_p_ = 0.38; Delay × Offload: *F* (2.07, 194.16) = 12.07, *p* < 0.001, η^2^_p_ = 0.11; Clock × Offload × Delay: *F* (2.07, 194.16) = 3.77, *p* = 0.024, η^2^_p_ = 0.04). After observing significant main effects associated with Delay and Offload, we conducted further exploratory analyses, which revealed that the main effects of Delay and Offload were significant in the hidden-clock group (Delay: *F* (2.35, 110) = 33.9, *p* < 0.001, η^2^_p_ = 0.06; Offload: *F* (1, 47) = 71.3, *p* < 0.001, η^2^_p_ = 0.21), but not in the persistent-on group (Delay: *F* (2.41, 113) = 2.25, *p* = 0.099, η^2^_p_ = 0.003; Offload: *F* (1, 47) = 0.10, *p* = 0.752, η^2^_p_ < 0.01). Then we conducted a series of post-hoc t-tests with a Bonferroni correction to compare performance across delay conditions in the hidden-clock group, and significant results emerged only when comparing the ‘no target’ condition with the other conditions (unaided: no-target vs. 10 s: *p* < 0.001, no-target vs. 20 s: *p* < 0.001, no target vs. 30 s: *p* < 0.001, 10 s vs. 20 s: *p* = 0.345, 10 s vs. 30 s: *p* = 0.810, 20 s vs. 30 s: *p* = 1.00; aided: no-target vs. 10 s: *p* < 0.001, no-target vs. 20 s: *p* = 0.016, no target vs. 30 s: *p* = 0.047, 10 s vs. 20 s: *p* = 0.277, 10 s vs. 30 s: *p* = 0.244, 20 s vs. 30 s: *p* = 1.00). The Delay × Offload interaction was significant only in the hidden-clock group (hidden-clock: *F* (1.89, 89) = 10.6, *p* < 0.001, η^2^_p_ = 0.02; persistent-on: *F* (2.37, 112) = 2.05, *p* = 0.125, η^2^_p_ < 0.01). Thus, participants in the hidden-clock group had worse performance (i.e. d-prime) than the persistent-on group. The *d’* of the hidden-clock group was significantly improved when they could use reminders, and the improvement was greater at longer delays. This suggests that offloading one task requirement to external reminders allowed participants to reallocate cognitive resources to a different aspect of the task, thereby improving their ability to perform it. This is consistent with the saving enhanced memory (Storm & Stone, 2014), saving enhanced performance (Runge et al., 2019) and cognitive spillover effects (Dupont et al., 2023) that have been reported in other studies of cognitive offloading. However, in the persistent-on group, neither offloading nor delay length had a significant effect on the *d*-prime of the 2-back task.

We also analysed the RT measure with within-subject factors, Offload (unaided, reminder), Match (match, nonmatch), and Delay (no-target, 10 s, 20 s, 30 s), as well as a between-subject factor, Clock (persistent-on, hidden-clock). Significant main effects were found for Delay (*F* (2.61,245.69) = 12.01, *p* < 0.001, η^2^_p_ = 0.11) and Match (*F* (1, 94) = 17.34, *p* < 0.001, η^2^_p_ = 0.16), but not for Offload and Clock (Offload: *F* (1, 94) = 0.25, *p* = 0.62, η^2^p < 0.01; Clock: *F* (1, 94) = 0.02, *p* = 0.887, η^2^_p_ < 0.01). The Delay × Clock (*F*(2.61, 245.69) = 3.61, *p* = 0.018, η^2^_p_ = 0.04) interaction was significant but the other interactions were not (Clock × Offload: *F* (1, 94) = 3.83, *p* = 0.053, η^2^_p_ = 0.04; Clock × Match: *F* (1,94) = 0.01, *p* = 0.931, η^2^_p_ < 0.01; Delay × Offload: *F* (2.64, 248.21) = 1.84, *p* = 0.148, η^2^_p_ = 0.02; Offload × Match: *F* (1, 94) = 0.07, *p* = 0.797, η^2^_p_ < 0.01, Delay × Match: (*F* (2.61, 245.55) = 1.18, *p* = 0.316, η^2^_p_ = 0.01). Response times were significantly shorter for match trials compared to nonmatch trials (hidden-clock: match: *M* = 665 ms, *SD* = 222; nonmatch: *M* = 703 ms, *SD* = 199; persistent-on: match: *M* = 669 ms, *SD* = 202; nonmatch: *M* = 709 ms, *SD* = 164). In the persistent-on group, participants responded to the ongoing task more quickly when there was no PM target (no-target: *M* = 670, *SD* = 176; 10 s: *M* = 687, *SD* = 194; 20 s: *M* = 696, *SD* = 185; 30 s: *M* = 704, *SD* = 184), while no such difference was noted in the hidden-clock group (no-target: *M* = 681, *SD* = 211; 10 s: *M* = 672, *SD* = 209; 20 s: *M* = 693, *SD* = 217; 30 s: *M* = 690, *SD* = 208). In conclusion, setting reminders in the PM task did not lead to faster responses in the 2-back task. Potentially, this was caused by the instructions that emphasised accuracy over speed. Furthermore, response times were shorter for target trials compared to non-target trials.
